# Altered brain function during movement programming is linked with motor deficits after stroke: a high temporal resolution study

**DOI:** 10.3389/fnins.2024.1415134

**Published:** 2024-08-12

**Authors:** Célia Delcamp, Alexandre Chalard, Ramesh Srinivasan, Steven C. Cramer

**Affiliations:** ^1^Department of Neurology, University of California, Los Angeles, Los Angeles, CA, United States; ^2^California Rehabilitation Institute, Los Angeles, CA, United States; ^3^Department of Cognitive Sciences, University of California, Irvine, Irvine, CA, United States

**Keywords:** biomarker, cortical activity, event-related desynchronization, motor control, premovement

## Abstract

**Introduction:**

Stroke leads to motor deficits, requiring rehabilitation therapy that targets mechanisms underlying movement generation. Cortical activity during the planning and execution of motor tasks can be studied using EEG, particularly via the Event Related Desynchronization (ERD). ERD is altered by stroke in a manner that varies with extent of motor deficits. Despite this consensus in the literature, defining precisely the temporality of these alterations during movement preparation and performance may be helpful to better understand motor system pathophysiology and might also inform development of novel therapies that benefit from temporal resolution.

**Methods:**

Patients with chronic hemiparetic post-stroke (*n* = 27; age 59 ± 14 years) and age-matched healthy right-handed control subjects (*n* = 23; 59 ± 12 years) were included. They performed a shoulder rotation task following the onset of a stimulus. Cortical activity was recorded using a 256-electrode EEG cap. ERD was calculated in the beta frequency band (15–30 Hz) in ipsilesional sensorimotor cortex, contralateral to movement. The ERD was compared over time between stroke and control subjects using permutation tests. The correlation between upper extremity motor deficits (assessed by the Fugl-Meyer scale) and ERD over time was studied in stroke patients using Spearman and permutation tests.

**Results:**

Patients with stroke showed on average less beta ERD amplitude than control subjects in the time window of −350 to 50 ms relative to movement onset (*t*(46) = 2.8, *p* = 0.007, Cohen’s d = 0.31, 95% CI [0.22: 1.40]). Beta-ERD values correlated negatively with the Fugl-Meyer score during the time window −200 to 400 ms relative to movement onset (Spearman’s *r* = −0.54, *p* = 0.003, 95% CI [−0.77 to −0.18]).

**Discussion:**

Our results provide new insights into the precise temporal changes of ERD after hemiparetic stroke and the associations they have with motor deficits. After stroke, the average amplitude of cortical activity is reduced as compared to age-matched controls, and the extent of this decrease is correlated with the severity of motor deficits; both were true during motor programming and during motor performance. Understanding how stroke affects the temporal dynamics of cortical preparation and execution of movement paves the way for more precise restorative therapies. Studying the temporal dynamics of the EEG also strengthens the promising interest of ERD as a biomarker of post-stroke motor function.

## 1 Introduction

Stroke is a leading cause of motor impairment worldwide, with a majority of patients experiencing upper extremity deficits (Rathore et al., 2002). An important direction for stroke rehabilitation targeting these deficits is to understand the mechanisms underlying movement generation. Electroencephalography (EEG) is a non-invasive tool useful for studying and measuring cortical activity during the planning and execution of a motor task by recording the associated electrical activity of the brain. Analysis of EEG data thereby allows for characterization of pathological patterns of cortical activation during movement by patients with stroke.

In humans, the initiation of a motor task is preceded within 500 ms by a slow decrease in EEG amplitude measured over the primary motor cortex. These potentials are known as motor related cortical potentials (MRCP) (Shakeel et al., 2015). MRCP are a consistent across a range of motor behaviors, e.g., being present during self-paced and cued movements (Shibasaki and Hallett, 2006).

Spectral modulations of cortical oscillations in the beta frequency band (13–30 Hz), such as beta event-related desynchronization (ERD), are particularly relevant to motor function and behavior. Cortical oscillations in the beta frequency band over the sensorimotor cortex are heavily involved in motor control. They are present at rest and decrease slightly before, then further during, a movement, and this decrease in beta power defines an ERD (Pfurtscheller and Lopes da Silva, 1999; Pfurtscheller, 2001). This ERD corresponds to increased corticospinal excitability and in most settings is interpreted as an electrophysiological correlate of cortical activations involved in the generation of movement (Takemi et al., 2013a,b). During the execution of a movement by a paretic limb, patients with stroke show an ERD in ipsilesional sensorimotor cortex, measured using EEG or magnetoencephalography, that has a smaller amplitude compared to that of healthy controls (Platz, 2000; Fu et al., 2006, p. 200; Gerloff et al., 2006; Rossiter et al., 2014; Park et al., 2016; Chalard et al., 2020). Some of these studies have studied movement preparation using *a priori* assumptions regarding choice of temporal window to study, for example, a window between −750 and −500 ms before movement onset (Platz, 2000) and the first 2 s before movement onset (Fu et al., 2006). Studies of the ERD may also be useful to understand how stroke affects motor system function, as ERD amplitude correlates with degree of motor deficits in the subacute (Bartur et al., 2019; Tang et al., 2020) and chronic phase and is reduced compared to healthy controls (Fu et al., 2006).

Defining, without any *a priori* assumptions, the exact timepoints during the genesis of movement when beta-ERD is altered by stroke, and is related to motor deficits, may be helpful to understand motor system pathophysiology and might also inform development of novel therapies (Fu et al., 2006; Norman et al., 2018), such as brain-machine interfaces and certain forms of brain stimulation. EEG has excellent temporal resolution and so is useful for understanding how alterations in brain function are related to deficits in motor status. We studied 27 patients with hemiparetic stroke and, as an initial step, confirmed that beta-ERD is reduced as compared to age-matched healthy controls, and that this reduction is proportional to the degree of motor deficits. In the current study, we sought to determine: (1) the time window during which beta-ERD differs between patients and controls, hypothesizing that we would observe a lower ERD amplitude for patients during a discrete epoch before movement and exploring whether this extends to the time period after movement is initiated; and (2) the time window during which the amplitude of beta-ERD is reduced in proportion to motor deficits after stroke, hypothesizing that timepoints when patients had reduced ERD compared to controls would be clinically relevant, i.e., that at timepoints with reduced ERD after stroke, ERD would also correlate with deficits in motor performance. In addition, an exploratory analysis examined functional connectivity, measured as EEG coherence, in relation to stroke and motor deficits, to better understand changes in ERD.

## 2 Methods

### 2.1 Subjects

Fifty adults (≥18 years old) consented to study participation. Entry criteria for all subjects were English-speaking, no disorder interfering with ability to follow study procedures, no coexisting diagnosis affecting arm function (apart from stroke), and no history of craniotomy. In addition, patients with stroke were required to have a history of unilateral hemiparetic stroke >30 days prior that included upper extremity weakness, defined as score ≤ 61 on the upper extremity motor Fugl-Meyer (UE-FM) (See et al., 2013) scale. Healthy control subjects were age-matched to patients with stroke.

### 2.2 Behavioral testing and neuroimaging

Testing in all subjects included the Nottingham Sensory Test (maximum 11 points, higher is better), Geriatric Depression Scale (maximum 15 points, lower is better), and Montreal Cognitive Assessment (MoCA; maximum 30 points, higher is better). In addition, patients with stroke were tested on the UE-FM (maximum 66 points, higher is better), modified Rankin Scale (mRS; lower is better), and Line Cancelation Test (higher is better). Assessors were certified on the UE-FM and MoCA scales.

Neuroimaging was retrieved from past medical records whenever possible. Images were reviewed for stroke location, subtype (ischemic vs. intracerebral hemorrhage), and involvement of primary motor cortex (M1). Lesion volume was measured by hand using MRIcron.^1^

### 2.3 Standard protocol approvals, registrations, and subject consents

This study was approved by the institutional review board of the University of California Irvine. All subjects gave written informed consent at their entry into the protocol.

### 2.4 EEG acquisition

Subjects were fitted with a 256-electrode EEG cap (HydroCel Sensor Net, Electrical Geodesics, Inc., Eugene, OR, United States). The EEG data were sampled at 1,000 Hz using a high-input impedance Net Amp 300 amplifier (Electrical Geodesics, Inc.) and NetStation 4.5.3 software. The EEG signals were referenced to the vertex electrode (Cz) during recording. The inputs from the splint apparatus were recorded by the EEG amplifier at 1,000 Hz. The onset of the stimulus was recorded by the EEG amplifier using a light sensor (Cedrus, San Pedro, CA, United States).

### 2.5 EEG experimental task

**Figure 1:**
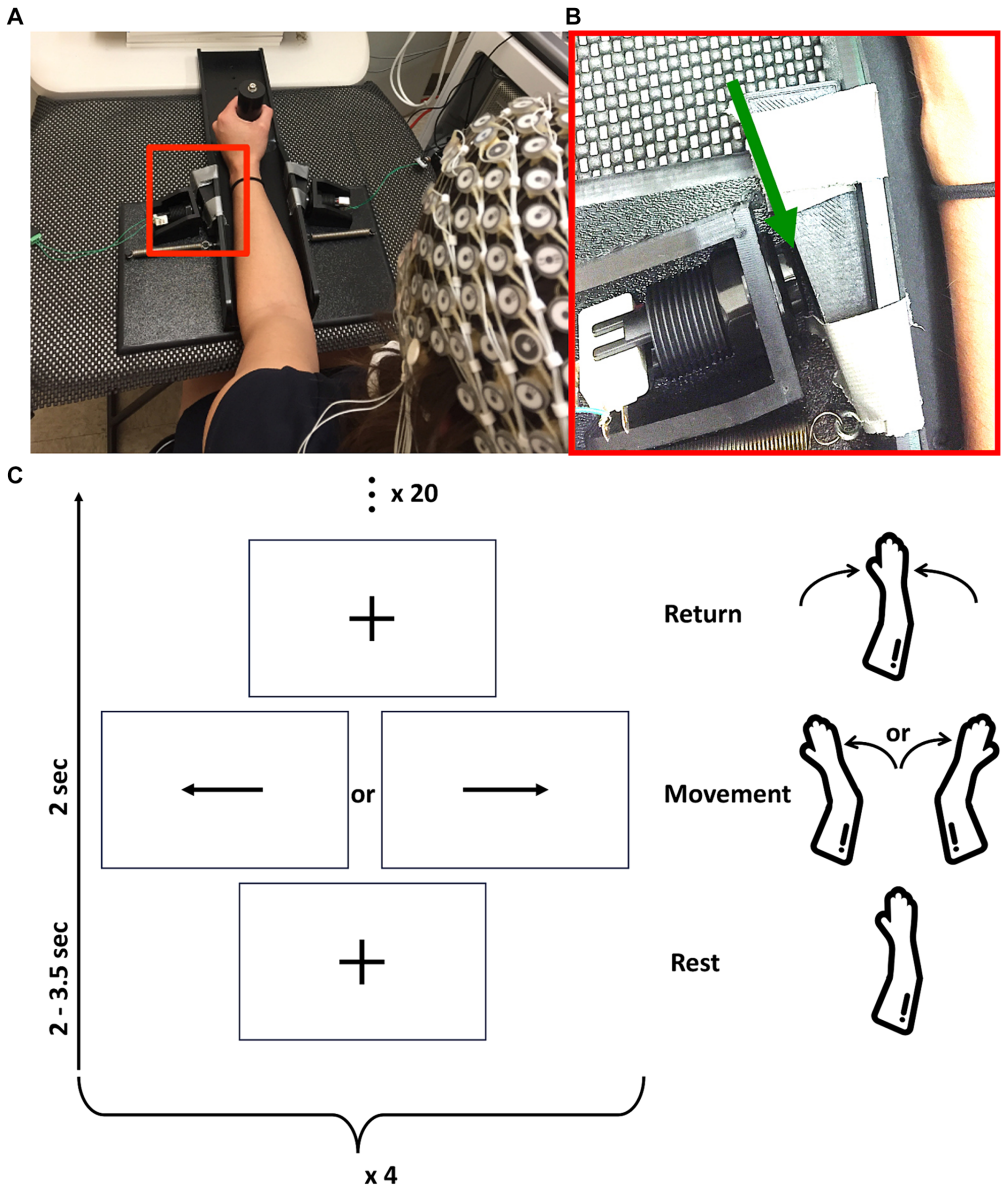
**(A)** Rest position of subjects with forearm in the tabletop splint. **(B)** Enlargement of the red box from **(A)** (with contrast adjusted for visibility), with the green arrow pointing to the 2 mm gap between splint and switch. This allows movement of the forearm in the plane of the table, after which the splint contacts the switch and can be displaced no further. In this image, the subject’s forearm is on the right side of the image and a switch is on the left side. **(C)** Representation of study design composed of four blocks of 20 movements. Each movement starts with rest, followed by movement cue, followed by return of the arm to the basal position, as outlined in vertically ascending order.

Subjects performed a task that required evaluation of a visual stimulus and a motor response. Each subject sat on a chair facing a monitor, with their back against the backrest, hips and knees at approximately 90°, and forearm (for stroke patients, paretic forearm; for healthy controls, right forearm) in a splint. This plastic splint sits on the proximal aspect of the table, moves with very low friction (~1 N resistance), and limits forearm movement to internal or external rotation at the shoulder in the plane of the tabletop (Figure 1A). During each trial of the task, subjects focused visually on a fixation cross until a stimulus (an arrow facing right or left) appeared; it then remained on the screen for 2 s. To obtain a self-initiated movement while controlling the inter-stimulus rest time, subjects were instructed to initiate the motor response freely following the onset of the stimulus. The motor response was given by internal or external (depending to the direction of the arrow) shoulder rotation of 2–3°, corresponding to 2 mm of hand displacement. This rotational motion triggered a mechanical motion detector for either direction of movement, which allowed for recording of the time at which the motion started (Figure 1B). Response time was defined as the time between stimulus onset and movement initiation. After each movement, subjects received verbal instruction to return their arm to the initial (mid) position of the apparatus, which was verified by the examiner. The rest period between the return to the initial position and the next stimulus was jittered between 2 and 3.5 s, to avoid cue anticipation. Participants completed four blocks of this task, with 20 trials/block (Figure 1C). After each block, participants received a 30s rest break during which the screen was blank. The order of internal and external shoulder rotation movements was counterbalanced and combined in analyses.

### 2.6 EEG preprocessing

Continuous EEG data were resampled at 200 Hz, high pass filtered at 0.5 Hz, and a 60 Hz notch filter was applied. EEG data were preprocessed using the EEGLAB clean_rawdata (Delorme and Makeig, 2004) pipeline: first, channels with artifact were removed, using the automatic channel rejection algorithm of the EEGLAB clean_rawdata plugin. Flat channels (having no signal variation over 5 s), noisy channels (signals with no variation over 5 s), and channels poorly correlated with other channels were detected, rejected, and interpolated using a spline interpolation. Second, artifacts were corrected using the EEGLAB ICLabel plugin. The EEG data were decomposed by independent component analysis using the EEGLAB runica function, then ICLabel classified independent components as artifact or EEG based on a supervised learning algorithm considering features in the spatial, spectral, and temporal domain. Independent components considered to be eye, muscle, or heart artifacts with a probability of at least 80% were removed, and their activities were subtracted from the data.

The EEG data were then segmented into 3,500 ms trials, each ranging from −2000 to +1,500 ms around stimulus presentation. Cutting at +1,500 ms after the stimulus enabled retention of all movements of even the slowest subjects without cutting into the return to initial position phase of the fastest subjects. To remove bad EEG epochs, a data-driven segment rejection approach based on quality assessment metrics was used. For each EEG epoch, epochs of high amplitude, timepoints of high variance, and channels of high variance were identified (Pedroni et al., 2019). Based on these metrics, epochs in the 90th percentile or greater regarding high amplitude, timepoint variance, and channel variance were rejected. To improve the spatial distribution of EEG signals, surface Laplacian was applied on the EEG data (Perrin et al., 1989). Finally, to enable comparison of patients with stroke to healthy controls, EEG data of stroke patients with right brain lesions were flipped along the midline to the left brain. As a result, C3 is always contralateral to movement: C3 is over the ipsilesional hemisphere for all patients with stroke and it is over the left hemisphere for healthy controls.

### 2.7 Event-related desynchronization

The ERD was calculated using time-frequency analysis [using a Morlet wavelet (Grinsted et al., 2004)]. The scale resolution of the wavelet (parameter “nvoice”), the number of scales used in the wavelet analysis (parameter “J1”), and the Morlet mother wavelet parameter (parameter “wavenumber”) were set to 7, 20, and 10, respectively. The ERD was obtained using the method described by Pfurtscheller (2001), whereby a decrease in power corresponds to an ERD, calculated using the following formula:


$$ERD\left(t\right)=\left[\left(A\left(t\right)-B\right)/B\right]\;*100.$$

where A is the absolute power at time t, and B is the mean of the absolute power during a baseline period ranging from −2000 to −1500 ms before stimulus onset. The ERD is therefore calculated as the power at each instant relative to the baseline power (X 100). In the current work our analysis was focused on the ERD in the beta (15–30 Hz) frequency band in leads approximately overlying ipsilesional M1 (Homan et al., 1987). Values of ERD reported represent the average across seven leads: it is the mean ERD in C3 as well as the mean ERD in the six leads that immediately surround C3. Data processing and statistical analysis were performed with the MATLAB software [version: 9.13.0 (R2022b)].

### 2.8 Cortical coherence

Cortical coherence was calculated for three signals of interest: (1) C3-F3, (2) C3-P3, and (3) C3-C4.

For each movement, spectral analysis was performed using a wavelet transform with the same characteristics as for the ERD analysis. The cross-spectrum between each signal of interest was calculated, after which cortical coherence was calculated by normalizing the cross-spectrum by the product of the auto-spectrum following formula:


$${R^{2}}_{\frac{EEG1}{EEG2}}\left(\omega ,u\right)=\frac{{\left|S_{\frac{EEG1}{EEG2}}\left(\omega ,u\right)\right|}^{2}}{S_{EEG1}\left(\omega ,u\right)S_{EEG2}{\left(\omega ,u\right)}^{\prime }}$$

where EEG1 and EEG2 are EEG time series, S_EEG1/EEG2_(ω, u) is the wavelet cross-spectrum between EEG time series at frequency ω and time u, and SEEG1(ω, u) and SEEG2(ω, u) are wavelet auto-spectra of EEG time series at frequency ω and time u (Delcamp et al., 2023).

A map of significant coherence was calculated using a binary mask of significance determined from the cross-spectrum and then applied to the coherence map to avoid retaining false positive values (Bigot et al., 2011). Coherence was then averaged in the beta frequency band (20–30 Hz) during two time windows: the premovement time window (from stimulus to start of movement) and the movement execution time window. Coherence during baseline was subtracted from that during premovement and movement, constituting the new coherence variables of interest.

### 2.9 Statistical analysis

Response time was compared between stroke patients and healthy controls. Data could be transformed to a normal distribution and so Student’s *t*-test was used.

The amplitude of the premovement beta-ERD was compared between stroke patients and healthy control subjects at each timepoint using a non-parametric permutation approach. This method provides a framework to enable statistical comparisons between entire time series data rather than imposing data reduction (Friston, 2007). Independent *t*-tests between stroke and healthy control groups were computed at each point in the time series, thereby forming a test statistic continuum. Next, groups of participants were randomly permuted, and the previous step for each permutation was repeated (*n* = 10,000) in order to generate random test statistic continuum distributions. To control for multiple comparisons, the test statistic continuum was compared to the random distribution given by the permutation analysis. If the statistic continuum exceeded the critical threshold (*α* = 0.05) given by the statistic continuum derived from the permuted data, a significant difference was deemed to exist. This procedure obviated concern for multiple comparisons while offering greater sensitivity than a conservative Bonferroni procedure (Maris and Oostenveld, 2007).

To better understand factors influencing beta-ERD after stroke, its amplitude was examined in the time window significantly different between groups in relation to key measures: side of stroke, lesion volume, lesion location, M1 injury, time post-stroke, Nottingham Sensory Test score, Geriatric Depression Scale score, and Montreal Cognitive Assessment score. These measures were not normally distributed, and so Mann–Whitney tests and Spearman’s rank correlation coefficient tests were used. The effect of M1 injury could not be disentangled from stroke location (see section 3.2), and so the critical thresholds were Bonferroni adjusted for seven comparisons, i.e., using *α* = 0.007 for these tests.

The amplitude of the beta-ERD was compared with the UE-FM score in patients with stroke using a non-parametric approach to assess correlation temporality. First, the Spearman’s rank correlation coefficient was calculated at each point in the time series, forming a correlation continuum. Next, groups of stroke patients were randomly permuted, and the previous step for each permutation was repeated (*n* = 10,000) in order to generate random correlation continuum distributions. Finally, the correlation continuum was compared to the random distribution given by the permutation analysis. If the correlation continuum exceeded the critical threshold (*α* = 0.05) given by the random distribution, a significant correlation was deemed to exist.

To better understand ERD findings, exploratory analysis examined three aspects of cortical coherence. First, values in patients were compared with those of healthy control subjects, for each of the three regions of interest, both premovement and during-movement; these were compared using Mann–Whitney tests, and significance for these six comparisons was set at *α* = 0.008. Second, in patients with stroke, each of the six measures of cortical coherence was compared with motor deficits (UE-FM score) using Spearman’s rank correlation tests at *α* = 0.008.

Sample size was calculated based on ability to detect a difference between groups in b-ERD with a SD that is 1.2-times larger than the difference between groups, assuming alpha = 0.05 at 80% power, yielding 23 subjects in each group; four extra patients with stroke were recruited in anticipation of subject dropout.

## 3 Results

**Figure 2:**
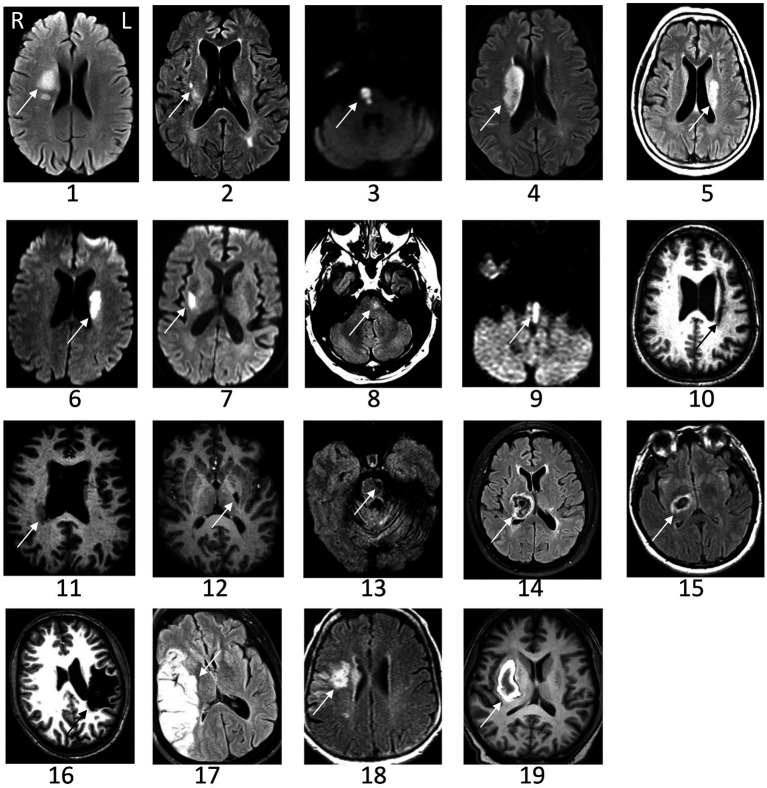
Representative images of the infarct are shown for patients with stroke. The arrow indicates the lesion. Images could not be retrieved from outside medical records in eight patients. L, left; R, right.

Subjects were studied from July 2018 to June 2019. Individual characteristics of patients with stroke appear in Table 1. Results are presented as mean ± SD for data following a normal distribution and median [1st Quartile–3rd Quartile] for data not following this distribution. All subjects were right-handed, except for two stroke patients who were left-handed and two who were ambidextrous. Patients were a median of 27 [4–78] months post-stroke. The paretic side was right in 14 and left in 13 patients. ERD variation curves over time and clinical parameters were similar between patients with lesions infarcts on the right and left side of the brain, and so patients were analyzed as a single group. The mRS score was 2.1 ± 0.8. The UE-FM score was 48 [41.5–57]. No patient with stroke had hemispatial neglect, as none made an error on the Line Cancellation Test. Brain imaging was available in 19 patients, and infarct volume was 13 [4.2–40] cc. Representative images of each infarct appear in Figure 2.

**Table 1:** Subject characteristics.

Patient	Age (yrs)	Sex	Nottingham sensory test	Geriatric depression scale	Montreal cognitive assessment	Upper extremity Fugl-Meyer	Time post-stroke (months)	Affected side of brain	Stroke subtype	Stroke location	M1 injured?	Infarct volume (cc)
1	61	F	11	2	21	57	4	Right	Isch	Sub	no	34.1
2	62	M	11	3	26	57	6	Right	Isch	Sub	no	1.4
3	62	M	11	0	26	30	9	Right	Isch	Sub	no	4.3
4	55	F	9	7	25	48	16	Right	Isch	Sub	no	40.1
5	59	F	11	11	27	45	5	Left	Isch	Sub	no	8.6
6	78	M	10	1	24	41	1	Left	Isch	Sub	no	10.0
7	65	M	11	2	29	46	2	Right	Isch	Sub	no	8.8
8	53	M	11	2	28	61	89	Left	Isch	Sub	no	1.1
9	75	M	10	3	27	53	45	Left	Isch	Sub	no	2.1
10	78	F	11	6	20	58	169	Left	Isch	Sub	no	19.9
11	65	M	1	4	29	26	39	Right	Isch	Sub	no	13.0
12	67	M	10	0	27	25	210	Left	Isch	Sub	no	3.3
13	40	F	2	3	23	21	44	Left	Hem	Sub	no	4.1
14	56	M	11	3	30	56	16	Right	Hem	Sub	no	40.1
15	29	F	11	1	30	37	27	Right	Hem	Sub	no	25.7
16	32	F	9	4	23	39	1	Left	Isch	Sub + Cort	yes	65.6
17	47	M	4	3	23	60	57	Right	Isch	Sub + Cort	yes	498.5
18	74	F	9	1	29	49	4	Right	Isch	Sub + Cort	yes	110.6
19	53	M	11	4	29	51	3	Right	Hem	Sub + Cort	yes	73.6
20	39	M	11	3	27	55	112	Right	*NA*	*NA*	*NA*	*NA*
21	49	M	11	3	29	43	40	Right	*NA*	*NA*	*NA*	*NA*
22	68	M	10	9	29	44	2	Left	*NA*	*NA*	*NA*	*NA*
23	66	M	11	1	28	58	6	Left	*NA*	*NA*	*NA*	*NA*
24	86	M	10	8	13	42	27	Left	*NA*	*NA*	*NA*	*NA*
25	66	M	11	4	28	60	33	Left	*NA*	*NA*	*NA*	*NA*
26	69	M	11	1	24	44	152	Right	*NA*	*NA*	*NA*	*NA*
27	51	M	7	2	21	59	76	Left	*NA*	*NA*	*NA*	*NA*

Values expressed as mean ± SD. Brain imaging was available in 19 patients. Isch, Ischemic stroke; Hem, Intracerebral hemorrhage; Sub, Subcortical stroke; Cort, Cortical stroke; M1, primary motor cortex; NA, Non-available.

Age did not differ between patients and controls (59 ± 12 vs. 59 ± 14, t_1,48_ = 0.13, *p* = 0.90, Cohen’s d = 0.04, 95% CI [−0.52: 0.59]). Patients had 19 males/8females, and controls had eight males/15 females (*χ*^2^ test: *p* = 0.01, odds ratio = 0.23, 95% CI [0.07–0.74]). The Nottingham Sensory test score was lower in patients compared to controls (11 [9.5–11] vs. 11 [11–11], Wilcoxon Signed Rank: *p* = 0.002; Rank-Biserial correlation = −1), indicating greater sensory deficits. The Geriatric Depression Scale was higher in patients compared to controls (3 [1.5–4] vs. 0 [0–1], Mann–Whitney *U-*test: *p* < 0.001, Rank-Biserial correlation = 0.76, 95% CI [0.58: 0.86]), indicating greater depression symptoms that on average did not meet criteria for major depression. The Montreal Cognitive Assessment score did not differ between groups (27 [23.5–29] in stroke vs. 27 [26–28] in controls, Mann–Whitney *U-*test: *p* = 0.72, Rank-Biserial correlation = −0.06, 95% CI [−0.37: 0.26]).

### 3.1 No difference in response time between groups

The response time following stimulus onset was not different between patients with stroke and healthy controls (626 ± 243 ms vs. 621 ± 383 ms, t_1,48_ = 0.23, *p* = 0.81, Cohen’s d = 0.06, 95%CI [−0.63: 0.49]).

### 3.2 Time window during which beta-ERD differs between patients and controls

**Figure 3:**
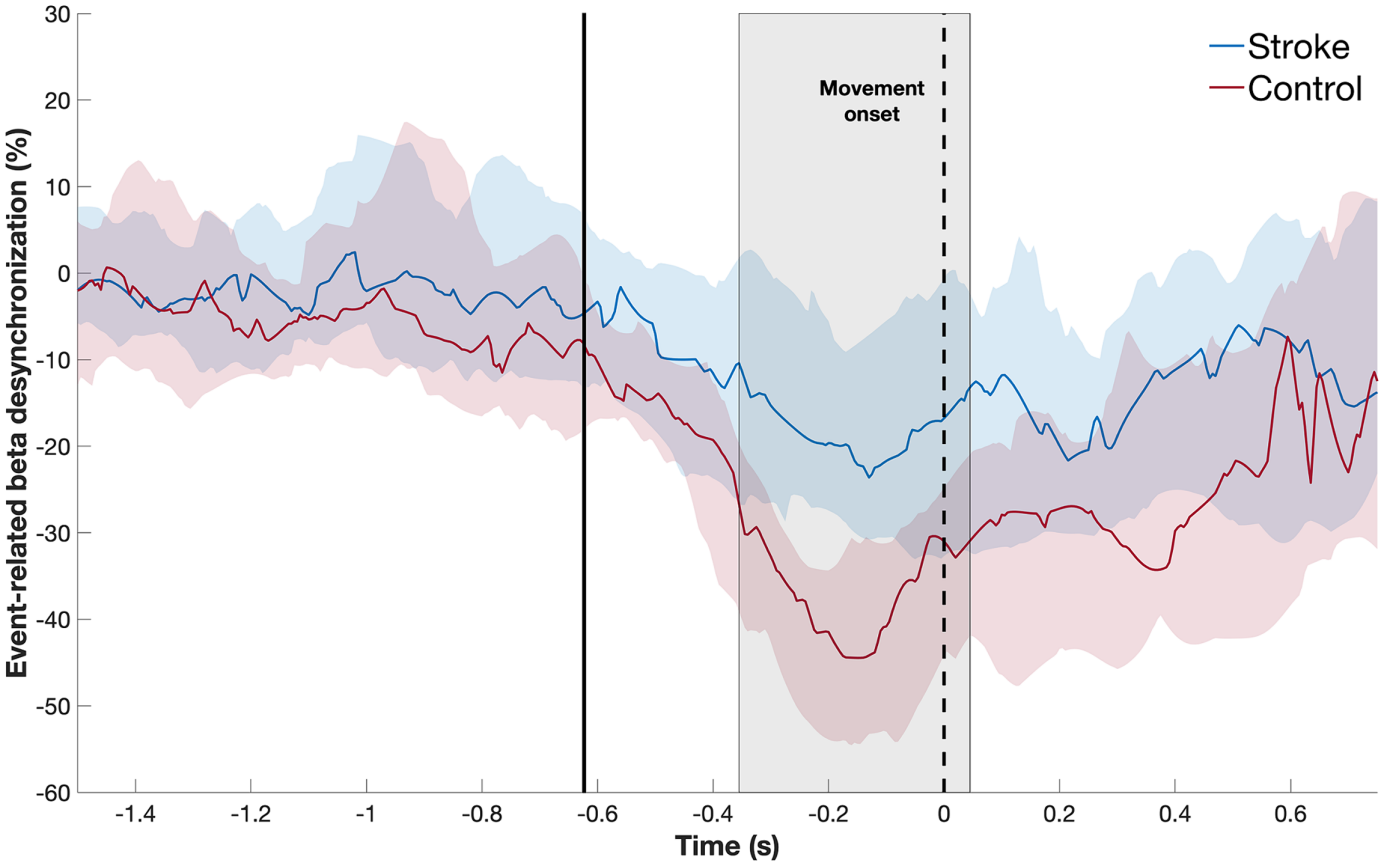
Temporal evolution of beta-ERD (blue line = stroke patients; red line = healthy controls) in C3 and surrounding leads. The shaded area represents the time window where the two subject groups show a statistically significant difference in amplitude, which occurred both before and during movement. The dotted black line indicates the start of movement; the solid black line, the average appearance of the stimulus.

**Figure 4:**
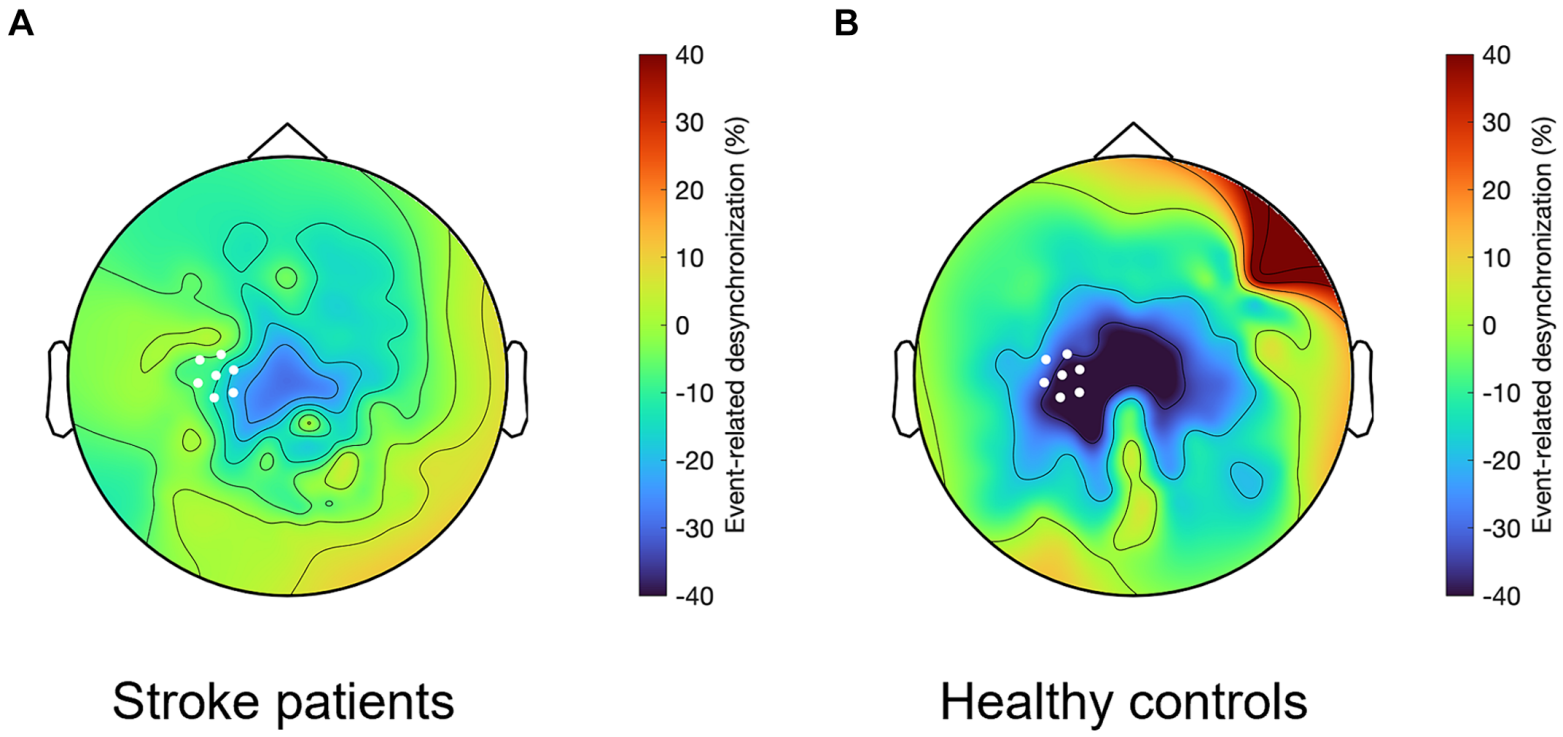
Topographical representation of the beta-ERD for the time window from −350 to +50 ms, which in **(A)** stroke patients shows a similar but smaller spatial distribution as compared to **(B)** healthy controls. White dots indicate C3 and the six leads surrounding it, which for patients with stroke is the ipsilesional hemisphere and for healthy controls it is the left hemisphere.

First, we defined the time window during which beta-ERD differs between groups, which was significant both before and during movement: patients with stroke, compared to healthy controls, showed smaller beta-ERD amplitudes in the time window of −350 to +50 ms relative to movement onset (gray box in Figure 3). We then compared the two groups with respect to the topographic distribution of beta-ERD during this time window. The spatial distribution in stroke patients is similar to that of healthy controls but occurs across a smaller number of leads (Figure 4).

**Figure 5:**
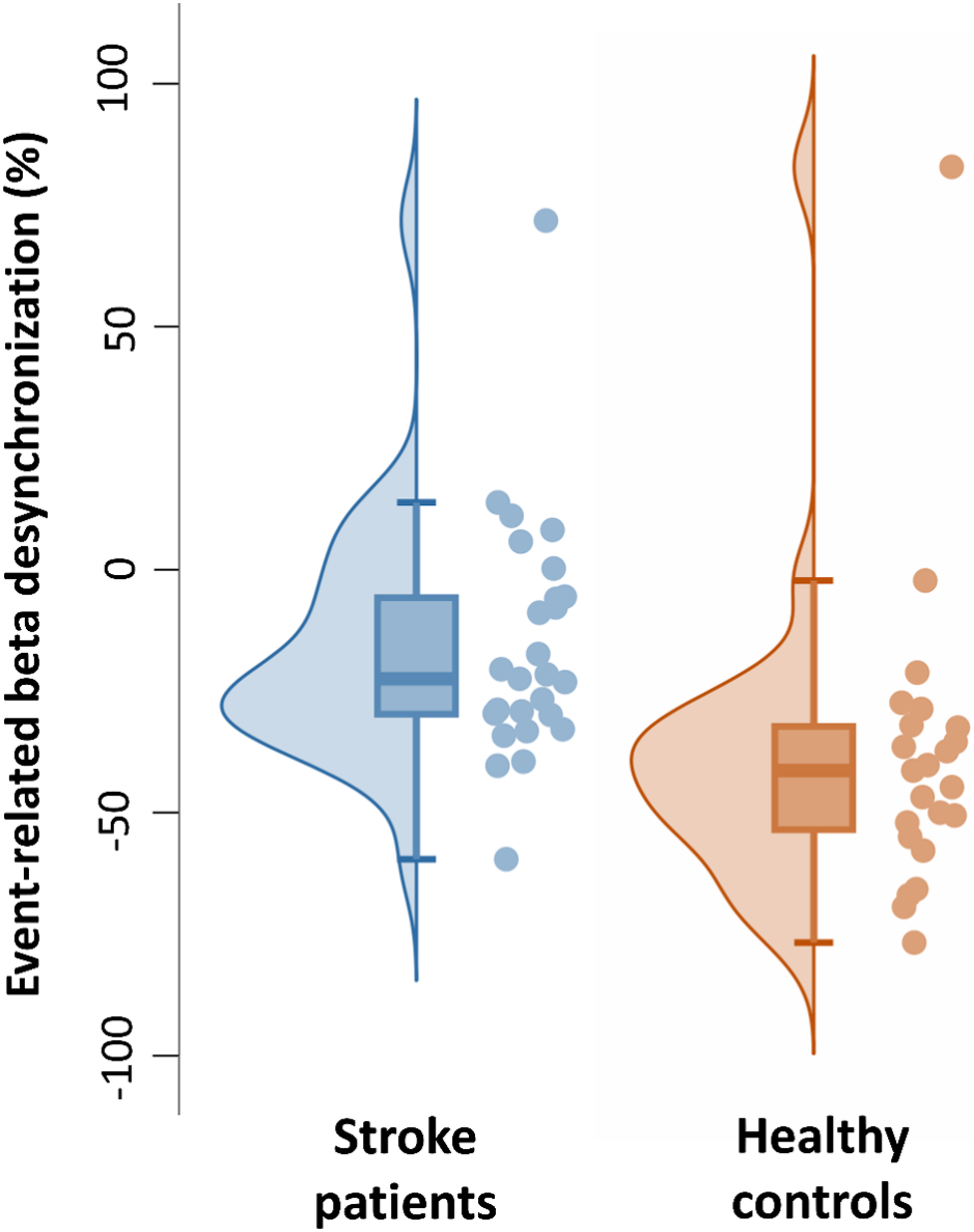
Individual and boxplot representation of the beta-ERD for the time window from −350 to +50 ms. In each box, the center line represents the median, the top, and bottom of the box correspond to the 25th and 75th percentiles, and the whiskers represent extreme values excluding outliers (<25% −1.5 interquartile range and > 75% +1.5 interquartile range). The amplitude of the beta-ERD was decreased in patients with stroke compared to age-matched healthy controls (Mann–Whitney *U-*test: *p* = 0.02, Rank-Biserial correlation = 0.39, 95% CI [0.09: 0.63]).

Next, we confirmed that, in this time window, the median amplitude of the beta-ERD was decreased in patients with stroke compared to age-matched healthy controls (Mann–Whitney *U-*test: *p* = 0.02, Rank-Biserial correlation = 0.39, 95% CI [0.09: 0.63]) (Figure 5). Outliers were defined as values lower than 25% −1.5 * interquartile range and higher than 75% +1.5 * interquartile range. One outlier was identified in each group. Excluding these outliers, the distribution of remaining values had a normal distribution, and so a *t*-test was used to compare the mean beta-ERD amplitude, which was decreased in patients with stroke compared to age-matched healthy controls (*t*(46) = 2.8, *p* = 0.007, Cohen’s d = 0.31, 95% CI [0.22: 1.40]). Note also that, in this time window, values for beta-ERD in patients with stroke did not differ according to side of stroke (Mann–Whitney *U-*test: *p* = 0.23, Rank-Biserial correlation = −0.29, 95% CI [−0.63: 0.15]), lesion volume (*p* = 0.55, Spearman’s r = 0.15, 95% CI [−0.38: 0.65]), lesion location (Mann–Whitney *U-*test: *p* = 0.96, Rank-Biserial correlation = −0.033, 95% CI [−0.60: 0.55]), time post-stroke (*p* = 0.60, Spearman’s *r* = 0.10, 95% CI [−0.31: 0.50]), Nottingham Sensory Test score (*p* = 0.36, Spearman’s *r* = −0.18, 95% CI [−0.58: 0.25]), Geriatric depression scale score (*p* = 0.84, Spearman’s *r* = 0.04, 95% CI [−0.29: 0.43]), or Montreal Cognitive Assessment score (*p* = 0.64, Spearman’s *r* = 0.09, 95% CI [−0.62: 0.49]). Patients with cortical stroke all had involvement of M1, and so the effect of M1 injury could not be disentangled from stroke location in the current cohort.

### 3.3 Time window during which the amplitude of beta-ERD is reduced in proportion to motor deficits after stroke

**Figure 6:**
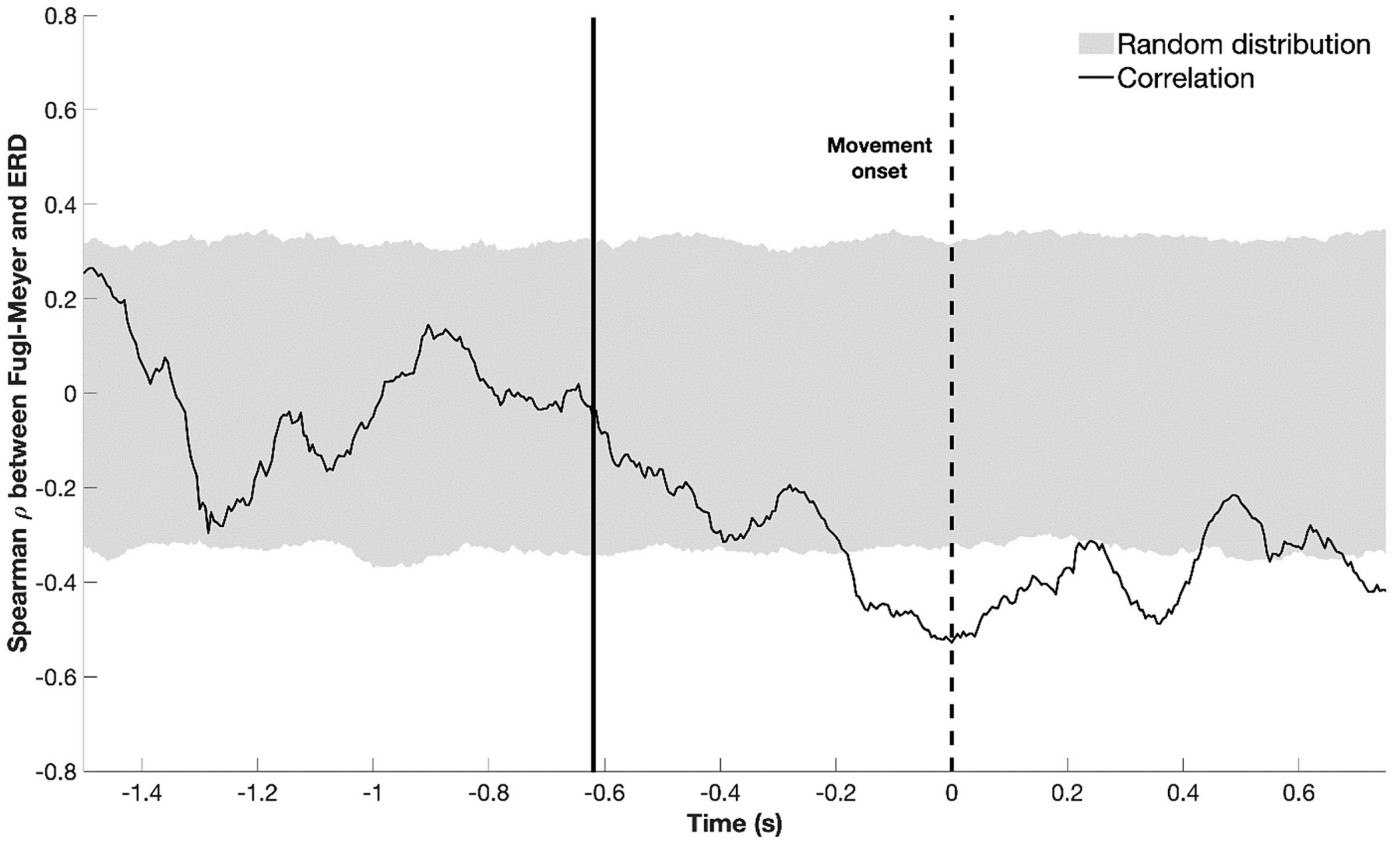
Temporal evolution of the correlation between beta-ERD and the upper extremity Fugl-Meyer score; gray shaded area represents a random distribution derived from permutation analysis, with values outside the gray box indicating a significant (*p* < 0.05) correlation. The dotted black line represents the start of movement; the solid black line, the average appearance of the stimulus. Negative values for ρ indicate that a more negative ERD correlates with higher UE-FM score—more cortical activity (a more negative ERD) is associated with better motor status (higher UE-FM score).

First, we defined the time window during which beta-ERD amplitude showed a significant relationship with motor deficits after stroke. An inverse correlation between beta-ERD and UE-FM score was significant from −200 to +400 ms relative to movement onset (Figure 6). This indicates that a healthier motor exam (smaller motor deficits) is associated with larger beta-ERD values both before and during movement.

**Figure 7:**
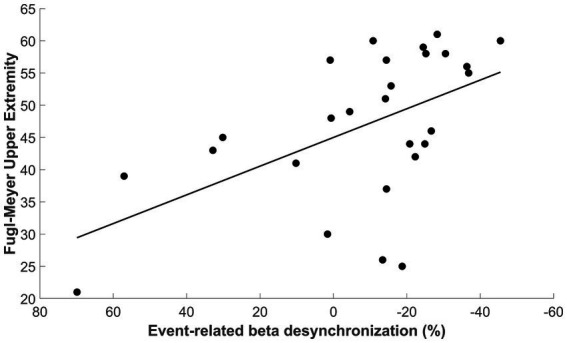
Correlation between beta-ERD for the time window from −200 to +400 ms and the upper extremity Fugl-Meyer score (Spearman’s *r* = −0.54, 95% CI [−0.77 to −0.18], *p* = 0.003).

Next, we confirmed that patients with a larger beta-ERD negative amplitude in the ipsilesional sensorimotor cortex (i.e., a more negative ERD) had better motor status (i.e., a higher UE-FM score), as depicted in Figure 7 (*p* = 0.003, Spearman’s *r* = −0.54, 95% CI [−0.77: −0.18]). As a secondary analysis, we assessed the relationship between beta-ERD and motor status during the time window when there was a significant difference in beta-ERD between stroke patients and controls (−350 to 50 ms); during this time window, a significant correlation remained was also found between FM-UE and ERD (*p* = 0.048, Spearman’s *r* = −0.38, 95% CI [−0.72: 0.046]).

### 3.4 Ipsilesional coherence after stroke

Coherence between ipsilesional M1 (C3) and ipsilesional parietal cortex (P3) was significantly different between subject groups, being smaller in stroke patients as compared to control subjects during premovement (−0.005 [−0.011–0.002] vs. −0.015 [−0.032 to −0.007], Mann–Whitney *U-*test: *p* = 0.002, Rank-Biserial correlation = 0.49, 95% CI [0.21: 0.70]). No other differences in cortical coherence (i.e., between C3-F3 and C3-C4) were observed between patients and controls (Mann–Whitney *U-*test: *p* > 0.38, Rank-Biserial correlation <0.15). No measure of cortical coherence was significantly related to motor deficits (Fugl-Meyer score) in patients with stroke.

## 4 Discussion

We aimed to map the time course of EEG changes before and during a visually cued motor task in patients with hemiparetic stroke. The results confirm that after hemiparetic stroke, the beta-ERD is decreased on average in amplitude compared to healthy controls, and it occurs across a more restricted cortical region as compared to healthy controls. Key new findings are that beta-ERD amplitude in patients with stroke is reduced as compared to healthy controls, on average, during a time window that spans premovement and movement execution epochs (specifically −350 to +50 ms), and that this decreased beta-ERD amplitude is proportional to extent of motor deficits during a specific time window (specifically −200 to +400 ms) that also extends from premovement to movement. Results support the utility of beta-ERD as a biomarker of motor system function after stroke and contribute to the understanding of how stroke affects the temporal dynamics of cortical preparation and execution of movement, which stands to inform emerging restorative therapies.

Beta-ERD was distributed bilaterally, though mainly in the hemisphere contralateral to movement. The reason for the observed bilateral distribution is not clear but may be due to the advanced age of participants, as higher age is known to be associated with a more bilateral distribution of motor function; such a change in motor function distribution is often seen in patients with stroke, who commonly have increased reliance on secondary motor areas during movement generation. We focused beta-ERD analysis on the ipsilesional hemisphere contralateral to movement, around C3, to ensure that current methods match those employed previously in the literature (Pfurtscheller et al., 1980; Stępień et al., 2011; Tangwiriyasakul et al., 2014; Ray et al., 2017; Pichiorri et al., 2018).

The amplitude of beta-ERD in ipsilesional M1 was, on average, diminished in patients with hemiparetic stroke, as compared to healthy controls, from −350 to 50 ms, with a peak difference around −200 ms relative to movement onset. This result is in line with other studies that showed that beta-ERD amplitude after stroke is reduced during the premovement period (Platz, 2000; Fu et al., 2006) and during movement (Gerloff et al., 2006; Rossiter et al., 2014; Park et al., 2016; Chalard et al., 2020), with current results providing details on the exact temporal dynamics of this relationship. Beta-ERD reflects decreased synchrony of neuronal populations (Pfurtscheller, 1992; Pfurtscheller, 2006) and is associated with increased neuronal excitability in thalamocortical networks (Steriade and Llinás, 1988), as excitatory modulatory inputs to motor cortex are increased even during anticipation of movement (Takemi et al., 2013a). Pathologically reduced beta-ERD amplitude may be associated with a decrease in control of movement (Tang et al., 2020). Overall, M1 coherence did not differ between patients and controls, with the sole exception being that coherence between ipsilesional M1 and parietal cortex was dampened in patients during movement execution. In the current study, reaction times by patients with stroke did not differ from those of healthy controls, though further assessment of motor control during EEG could not be performed given the motor task.

The amplitude of beta-ERD was reduced in proportion to motor deficits (Figure 7), a finding that is consistent with prior reports in subacute (Bartur et al., 2019; Tang et al., 2020) and chronic stroke (Fu et al., 2006). A key finding in the current report is that this relationship was present before and during movement, i.e., beta-ERD correlated with UE-FM score from −200 to +400 ms relative to movement onset (Figure 6). This time window covers the period from pre-movement to after the movement has started. Because this correlation appears in a time window where the desynchronization of beta power is still present (Figure 3), it is appropriate to label this as a correlation between the UE-FM score and beta-ERD. It is also reasonable to refer to this as a correlation between the UE-FM score and the power of beta band activity.” These findings highlight the value of studying the temporal evolution of beta-ERD in patients with stroke, as prior studies have generally focused only on timepoints following start of movement, but brain function during movement planning may also be important to understanding and treating motor deficits after stroke. Building on this, future studies might investigate ERD during a more complex motor task, such as one involving contraction with force feedback, as this would provide insights into the relationship that a wider range of motor behaviors, such as sensorimotor integration, have with underlying electrophysiological derangements. The beta-ERD amplitude in patients with stroke was related to UE-FM score but explained less than half of the variance (Figure 7) and did not differ according to side of stroke, time post-stroke, or the severity of sensory, depression, or cognitive deficits. The explanation may lie in the motor behaviors measured by the UE-FM scale, some of which (such as synkinesias) may arise from widely distributed cortical and subcortical circuits.

Sensorimotor cortex activity during movement can be bilateral in patients with stroke and in healthy control subjects (Park et al., 2016; Chalard et al., 2020; Williamson et al., 2022), but we did not observe a difference between groups in coherence between leads approximately overlying ipsilesional M1 and contralesional M1. However, we did observe a stroke-related decrease in ipsilesional coherence (i.e., between C3 and P3), similar to a prior report (Gerloff et al., 2006). The key novel finding here is that reduced coherence in patients with stroke was observed specifically during premovement, whereas the literature has mainly focused on motor system coherence during movement performance. Measures of coherence were not related to motor status (UE-FM score) in the current study. One prior study did find a positive correlation between ipsilesional coherence and motor status, as assessed by the UE-FM score (Zheng et al., 2022). Thus, while that prior study and the current one both found decreased ipsilesional coherence after stroke, differences were found as to whether this coherence was related to behavioral status; the basis for these divergent results is unclear but may relate to features of the study population or the EEG acquisition protocol.

The results support the utility of beta-ERD as a biomarker of sensorimotor cortex pathology for use in clinical trials of interventions that rely on high temporal resolution when aiming to improve motor status, including those that target the neural events underlying movement preparation. Features of the temporal evolution provides a broader picture of ERD than a single value reflecting the mean of the ERD in an investigator-selected time window of interest. A biomarker with high temporal resolution may prove useful for some restorative therapies. For example, the precision with which neural signals are sensed is critical for brain stimulation associated with a closed-loop brain-computer interface (Valenchon et al., 2022; Belkacem et al., 2023), and closed-loop non-invasive brain stimulation with millisecond precision enables selective interference with ongoing brain activity (Zrenner et al., 2016). Also, current findings may inform a strategy whereby learning to control electrical rhythms in sensorimotor cortices that occur prior to movement can improve finger extension ability for some patients with stroke (Norman et al., 2018). Some forms of non-invasive brain stimulation might also benefit from incorporation of a biomarker with high temporal resolution.

Limitations of the current study include the choice of task used to elicit the beta-ERD. We selected a simple, rapid, small amplitude, single-joint movement for this study; future studies may use a more complex movement requiring sensory feedback. The current patient population did not include patients in the initial days post-stroke, when post-stroke recovery is at a maximum (Duncan et al., 1992), and so attention to this detail can be incorporated into future study designs. When selecting healthy controls, our focus was on age-matching, which was achieved, however other behavioral variables differed between groups (Table 1), and this might have affected results. It is important to note, as illustrated in Figure 5, that although the median and mean beta-ERD (without outliers) differed between the two groups, the power of the effect was moderate (Cohen’s *d* = 0.31) and the beta-ERD of some patients was similar to that of some healthy control subjects. This report, therefore, does not identify pathological beta-ERD in all patients, but instead is focused on differences in group behavior. Finally, stroke is a very heterogeneous disease, and consistent with this the population of patients enrolled in this study varied with respect to factors such as time post-stroke and lesion characteristics, although these measures were not found to be related to ERD in the current patient cohort; future studies examining patients with more homogenous features may provide additional insights. Despite these limitations, the current study provides new insights into the effects of hemiparetic stroke on motor control and its link with motor impairments, findings that may be useful to development of novel therapies targeting stroke recovery and that inform use of EEG as a biomarker for clinical studies of novel therapies that rely on high temporal resolution.

## Data availability statement

Anonymized data will be made available by reasonable request from a qualified investigator.

## Ethics statement

The studies involving humans were approved by the institutional review board of the University of California Irvine. The studies were conducted in accordance with the local legislation and institutional requirements. The participants provided their written informed consent to participate in this study.

## Author contributions

CD: Formal analysis, Methodology, Writing – original draft. AC: Formal analysis, Methodology, Writing – original draft, Software, Visualization. RS: Formal analysis, Methodology, Software, Writing – original draft. SC: Formal analysis, Methodology, Writing – original draft, Conceptualization, Funding acquisition, Investigation, Resources, Supervision, Writing – review & editing.

## Footnotes

^1^ http://www.mccauslandcenter.sc.edu/mricro/mricron
